# Enhancement of neem growth and wood quality via humic acid and seaweed extract application under treated wastewater irrigation in arid environments

**DOI:** 10.1038/s41598-026-61599-z

**Published:** 2026-07-15

**Authors:** Sabri Salaheldin, Mohamed A. Selim, Wagdi Saber Soliman, Abd-Allah Gahory

**Affiliations:** Horticulture Department, Faculty of Agriculture and Natural Resources, Aswan University, Aswan, 81528 Egypt

**Keywords:** Arid regions, *Azadirachta indica*, Biomass productivity, Heavy metal translocation, Phytoremediation, Environmental sciences, Plant sciences

## Abstract

Wastewater-irrigated forestry is an important strategy for sustainable water resource management, biomass production, and ecosystem restoration in arid and semi-arid regions. A field experiment was conducted from September 2023 to June 2025 in Balana, Nasr El-Nuba District, Aswan Governorate, Egypt, to investigate the effectiveness of humic acid (HA) and seaweed extract (SWE) as biostimulants on the growth performance, physiological responses, heavy metal accumulation, and wood properties of *Azadirachta indica* irrigated with treated wastewater. The study followed a completely randomized block design with five treatments: control, HA at 1 and 2 mL L^−1^, and SWE at 1 and 2 g L^−1^. Measurements were recorded at 9 and 21 months after planting. Biostimulant application significantly enhanced vegetative growth, chlorophyll content, and wood quality compared with untreated plants. Seaweed extract at 2 g L^−1^ produced the greatest improvements in stem length by 7.7 and 11.3%, stem diameter by 35.4 and 10.7%, and chlorophyll content by 13.6 and 14.75 compared to control at 9 and 21 months, respectively. Also, it increased wood density by 10%, and reduced moisture by 9%. Whereas humic acid at 2 mL L^−1^ resulted in higher cellulose, hemicellulose, and holocellulose contents by 4, 6.4 and 10.2%, while reducing lignin and ash by 12.1 and 9.5% compared to control. Heavy metals (Pb, Cd, Cu, Fe, and Zn) accumulated in all plant organs, with accumulation patterns varying according to the element and biostimulant treatment. Most metals were preferentially retained in the roots, whereas Fe accumulated predominantly in the leaves, indicating different uptake and sequestration strategies. Despite continuous irrigation with treated wastewater, soil heavy metal concentrations declined over the experimental period, demonstrating the capacity of neem trees to remediate metals from contaminated soils. These findings demonstrate that integrating treated wastewater irrigation with biostimulant application can simultaneously improve tree productivity, wood quality, and environmental remediation. Seaweed extract at 2 g L^−1^ is recommended for maximizing tree growth and wood physical properties, while humic acid at 2 mL L^−1^ is preferable for improving wood chemical quality, highlighting the potential of this integrated approach for sustainable woody plantations in water-limited regions.

## Introduction

Water is fundamental to human civilization and remains essential for agricultural productivity, industrial development, and socio-economic stability^1^. However, rapid population growth—projected to reach 9.7 billion by 2050—has increased pressure on freshwater resources, making water scarcity one of the most critical environmental challenges of the twenty-first century^2^. Agriculture accounts for nearly 70% of global freshwater withdrawals, highlighting the need for sustainable water management strategies. One promising solution is the reuse of treated wastewater for irrigation, which conserves freshwater while enabling nutrient recycling in agricultural systems. In Egypt, wastewater production is expected to reach approximately 11.673 billion cubic meters by 2030, emphasizing its potential as an important non-conventional water resource^3^.

Alongside water reuse initiatives, afforestation has gained importance as a strategy for mitigating environmental degradation, restoring marginal lands, and enhancing carbon sequestration. Beyond ecological benefits, afforestation programs contribute to rural development, employment opportunities, and improved living standards, particularly in arid and semi-arid regions^4^. Assessing tree biomass and volume is essential for sustainable forest management, climate change mitigation, and ecosystem service assessment^5^. Therefore, selecting resilient and multipurpose tree species capable of thriving under harsh environmental conditions is essential for achieving environmental and economic sustainability.

Among such species, *Azadirachta indica* (neem) is a highly adaptable evergreen tree belonging to the Meliaceae family. Native to tropical and subtropical regions, neem grows well in diverse soil types, particularly sandy and deep soils, and tolerates extreme temperatures up to 47 °C without significant loss of bioactivity. The species is widely recognized for its cultural and economic importance, often referred to as the “village pharmacy” in India and the “Forty Tree” in Africa due to its numerous medicinal applications^6^. Its primary bioactive compound, azadirachtin, possesses strong pesticidal properties, making neem-based products widely used in organic agriculture, particularly in Europe. In addition, neem contributes to pharmaceutical, cosmetic, agricultural, and environmental industries^7^.

Heavy metals are major environmental pollutants primarily released through anthropogenic activities, including industrialization, fossil fuel combustion, urbanization, waste disposed, and agricultural practices^8,9^. Their accumulation in soils and groundwater poses significant ecological risks, as even low concentrations can impair plant growth by inducing oxidative stress, disrupting photosynthesis, interfering with essential metabolic processes, and accelerating senescence^10,11^.

Despite its adaptability, irrigation with treated wastewater may expose plants to residual concentrations of heavy metals and other contaminants that are not completely removed during the treatment process. In this context, phytoremediation has emerged as a sustainable method for mitigating pollutants through mechanisms such as phytoextraction, phytostabilization, phytodegradation, and phytovolatilization^10,12^. Plants, together with rhizospheric microorganisms, can absorb, transform, or immobilize contaminants, thereby improving soil and water quality^13^. However, the efficiency of phytoremediation is often limited by plant tolerance and environmental conditions.

To enhance plant resilience and productivity under such stress conditions, natural biostimulants have received increasing attention. Humic acid, a high-molecular-weight organic compound formed from the microbial decomposition of plant and animal residues, is abundant in soils and organic matter^14^. Humic acid improves soil structure, water-holding capacity, microbial activity, and nutrient availability, thereby promoting plant growth and physiological processes. However, its effectiveness may vary depending on its origin and chemical composition^15–17^.

Humic acid has recently gained considerable attention as an effective organic amendment for remediating contaminated soils. Humic acid fertilizers might help reduce the uptake of heavy metals by immobilizing heavy metals in the root zone and decrease their relative bioavailability^18^. It contains functional groups such as carboxyl, carbonyl, and quinone that interact with nutrients and heavy metals through chelation and adsorption processes^19^. The effectiveness of humic acid in remediating heavy metal-contaminated soils has been proved under various environmental conditions, including those in the Middle East. Previous research has reported significant reductions in heavy metal bioavailability following humic acid application under similar climatic conditions^20^. Furthermore, recent investigations have provided deeper insights into the interactions between humic acid and heavy metals, highlighting their role in regulating metal mobility and plant uptake mechanisms^21^.

Similarly, seaweed extracts derived from marine macroalgae are widely recognized as eco-friendly biostimulants rich in polysaccharides, phytohormones, vitamins, fatty acids, and mineral nutrients^22,23^. These extracts are considered sustainable alternatives to synthetic agrochemicals^24–26^. One of the most widely used species in commercial formulations is *Ascophyllum nodosum*, a brown alga native to the North Atlantic intertidal zones^27^. Its extracts typically contain carbohydrates, proteins, lipids, ash, and polyphenols that contribute to their growth-promoting effects^28^.

Seaweed extracts have beneficial effects on plant growth as well as improving their resistance to several biotic and abiotic stresses. Khattabi^29^ demonstrated that the aqueous extracts of *Fucus spiralis* and *Cystoseira ericoides* improved the plant response to heavy metals stress, highlighting the potential use of these seaweeds in phytoremediation processes. Recent studies have also documented seaweed extract ability to counteract the deleterious effects of pollutants on plants, thus increasing the phytoremediation efficiency of some species^30–32^. Given the increasing demand for sustainable water use and resilient afforestation systems in arid regions, integrating treated wastewater irrigation with natural biostimulants represents a promising strategy.

Previous studies mainly focused on vegetative growth and physiological responses of tree seedlings following biostimulant application, whereas little information exists on the effects of humic acid and seaweed extract on juvenile wood quality, including wood density, cellulose, lignin composition, and heavy metal accumulation under wastewater irrigation. Therefore, this study aimed to evaluate the effects of treated wastewater irrigation on the growth performance, biochemical composition, and wood properties of *A. indica* and to assess the effectiveness of humic acid and seaweed extract in enhancing tree growth and wood quality under these conditions.

## Materials and methods

### Experimental site and plant materials

The field experiment was conducted at a forest plantation of the wastewater irrigation facility in Balana, Nasr El-Nuba District, Aswan Governorate, Egypt, operated by the Aswan Drinking Water and Wastewater Company. One-year-old neem (*Azadirachta indica* A. Juss.) seedlings were obtained from the Tropical Farm, Kom Ombo, Aswan, Horticultural Research Institute, Agricultural Research Center, Egypt. Uniform seedlings (≈ 25 cm height and 0.5 cm stem diameter) were transplanted on 15 September 2023 at a spacing of 5 × 5 m, with ten seedlings per plot.

Before and 21 months of planting, soil samples (0–30 cm depth) represented the experimental plot, regardless of the biostimulant treatments, were collected and analyzed (Table 1). In addition, irrigation water were collected and analyzed for physical, chemical, and heavy metal characteristics (Table 2) according to standard procedures^33,34^. Soil texture was sandy with pH 7.87 and Ec of 1.4 dS m^−1^. The experiment was conducted at a governmental research site officially designated for treated wastewater irrigation, and all experimental activities were performed under the authorization of the Aswan Drinking Water and Wastewater Company.

**Table 1:** Heavy metals concentrations (mg kg^−1^) of soil before and 21 months after planting.

Element	Pb	Cd	Cu	Fe	Zn
Before planting	7.16	0.417	23.7	31558	54.6
21 months after planting	4.15	0.322	6.5	28190	29.2

**Table 2:** Physical, chemical, and heavy metal properties of studied irrigation water compared to Egyptian standard (Code 501/2015).

Parameter/ Element	Balana Value	Egyptian Limit	Status
Total Dissolved Solids	760 mg L^−1^	< 2000 mg L^−1^	✓ Acceptable
Total Suspended Solids	48 mg L^−1^	< 50 mg L^−1^	✓ Acceptable
Biochemical Oxygen Demand	38 mg L^−1^	< 40 mg L^−1^	✓ Acceptable
Chemical Oxygen Demand	70 mg L^−1^	< 100 mg L^−1^	✓ Acceptable
pH	8.2	6.5–8.5	✓ Acceptable
Lead	0.004 (mg L^−1^)	< 5 (mg L^−1^)	✓ Safe
Cadmium	0.002 (mg L^−1^)	< 0.01 (mg L^−1^)	✓ Safe
Copper	0.177 (mg L^−1^)	< 0.2 (mg L^−1^)	✓ Safe
Iron	4.354 (mg L^−1^)	< 5.0 (mg L^−1^)	✓ Safe
Zinc	3.480 (mg L^−1^)	< 5.0 (mg L^−1^)	✓ Safe

### Experimental design and the sources of used bio-stimulants

The experiment was conducted over two growing seasons (15 September 2023–15 June 2025) Using a completely randomized block design with five treatments and three replicates. The treatments consisted of: (1) control (no amendment), (2) 1 mL L^−1^ humic acid, (3) 2 mL L^−1^ humic acid, (4) 1 g L^−1^ seaweed extract, and (5) 2 g L^−1^ seaweed extract.

Humic acid (Canada Humax) was supplied by the Egyptian Canadian Company for Humate Technology and Agricultural Consultancy. According to the manufacturer’s specifications, the product contains 5% total potassium, present as potassium fulvate and potassium humate. The seaweed extract was derived from marine algae. Amendments as soil drenches (50 mL per seedling) were applied directly around the seedling root zone five times annually from March to June (2024 and 2025) at three-week intervals.

### Growth and physiological parameters

Measurements were performed 9 months (first season) and 21 months (second season) after transplanting. Three plants were randomly selected from each plot to determine stem length, stem diameter, leaf thickness, leaf relative water content (RWC), and total chlorophyll content.

Leaf thickness was estimated from leaf volume and leaf area determined using displacement method and ImageJ software, respectively. Leaf thickness has been calculated according to the following equation:

$$\:Leaf\:thickness=\frac{Leaf\:volume}{Leaf\:area}$$

Relative water content was calculated according to Zhou et al.^35^. Relative water content was calculated as following:

$$\:RWC=\frac{FW-DW}{TW-DW}\times\:100$$

as FW, DW, and TW represent fresh weight, dry weight, and turgid weight, respectively.

Total chlorophyll content was estimated using a SPAD-502 Plus chlorophyll meter and converted to chlorophyll concentration using the calibration equation of Dash et al.^36^:

$$\:Total\:chlorophyll\:\left(mg\:{m}^{-2}\right)=0.118{\mathrm{x}}^{2}+0.919x+7.925$$

Where; x represented SPAD value.

### Heavy metal analysis

Dried leaf sampled were wet digested using a nitric acid-perchloric acid mixture following the standard procedures^37,38^. Concentrations of lead (Pb), cadmium (Cd), copper (Cu), iron (Fe), and zinc (Zn) were determined by atomic absorption spectrophotometer using flame and graphite furnace atomization, depending on the element concentrations, following U.S. Environmental Protection Agency^38^ recommendations.

### Wood physical and chemical properties

At the end of the experiment (21 months), three trees per plot were destructively sampled for wood analyses. Ten wood specimens representing sapwood and heartwood were prepared from each sample for laboratory evaluation.

#### Moisture content (%),Wood density (g cm^−3^), and ash (%)

Wood moisture content was determined according to Desch and Dinwoodie^39^. Samples were oven-dried at 103 ± 2 °C to a constant weight, and moisture content was calculated as:

$$\:Moisture\:content\:\left(\%\right)=\frac{\mathrm{M}\mathrm{i}-\mathrm{M}\mathrm{o}}{\mathrm{M}\mathrm{i}}\times\:100$$

where Mi is the initial weight (g) and Mo is the oven-dry weight (g).

Wood density was determined following the method described by Haygreen and Bowyer^40^ based on the air-dried weight (12–14% moisture content) and corresponding volume measured by the water displacement method. Density was calculated as:

$$\:D\:\left(g\:{cm}^{-3}\right)=\frac{W}{{V}_{b}-{V}_{a}}$$

where W is the air-dried weight (g), Vb is the water volume before immersion (cm^3^), and Va is the water volume after immersion (cm^3^).

Each air-dried wood was weighed to obtain dry weight and then inserted into a digital muffle furnace (F-27, Carbolite, Shfld.) set at 525 °C for 6 h to determine the ash content (%).

### Determination of cellulose, holocellulose, and hemicellulose contents (%)

Cellulose content was estimated following the method of Sadasivam and Manickam^41^. Finely powdered, pre-extracted sawdust samples were analyzed using the anthrone colorimetric method, and absorbance was measured at 630 nm using a spectrophotometer. Cellulose concentration was calculated using a standard curve prepared using known cellulose standards.

Holocellulose content was determined according the sodium chlorite method of Erickson^42^. Oven-dried, extractive-free wood samples were delignified under acidic conditions, and the holocellulose fraction was recovered, dried to constant weight, and expressed as a percentage of the oven-dry sample weight. Hemicellulose (%) content was calculated as the difference between holocellulose and cellulose contents:

$$\:Holocellulose\:\%=\frac{Dry\:residue\:weight\:}{Initial\:oven-dry\:weight}\times\:100$$

$$\:Hemicellulose\:\%=Holocellulose\:\%-cellulose\:\%$$

### Determination of lignin content (%)

Lignin content was determined following the TAPPI T222 om-02 standard method^43^. Extractive-free, oven-dried wood samples were hydrolyzed with sulfuric acid, and the acid-insoluble lignin residue was recovered and oven-dried to constant weight. Lignin content was expressed as a percentage of oven-dry sample weight.

### Statistical analysis

Experimental data were subjected to statistical analysis using the F-test as described by Snedecor and Cochran^44^ to evaluate treatment significance. Differences among the treatments were assessed using Tukey’s honestly significant difference (HSD) test at 5% level, following the procedure described by Gomez and Gomez^45^. The values represent the mean of independent replicates ± standard deviation. Statistical analysis was performed using JMP software (versions 16.0; SAS Institute Inc., USA).

## Results

### Growth and physiological parameters

**Fig. 1:**
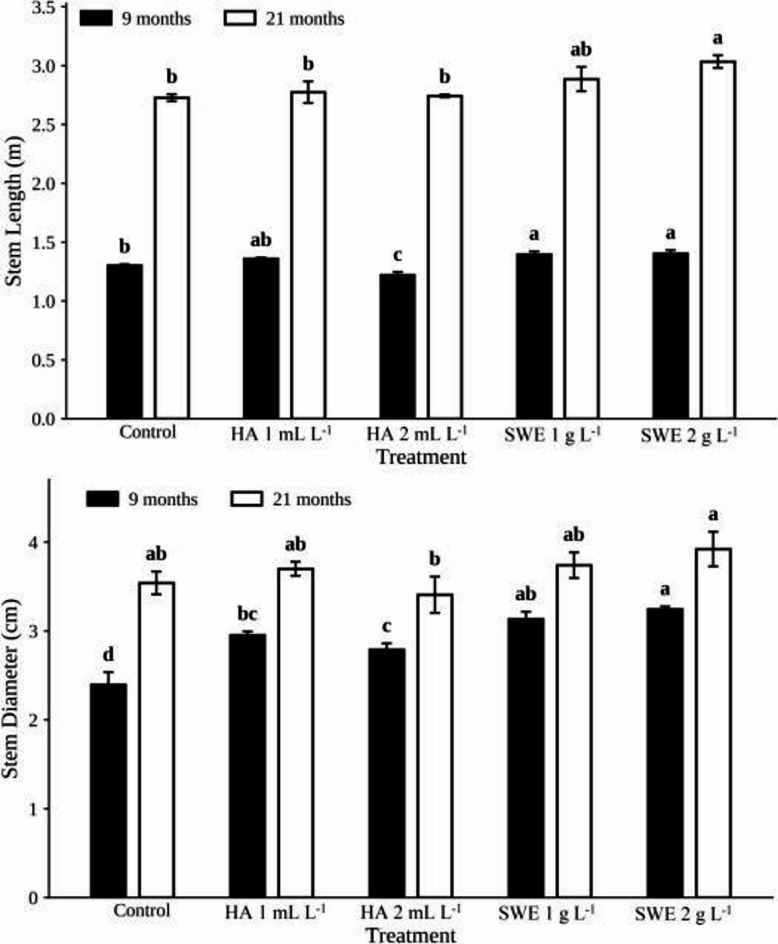
Effect of humic acid (HA) and seaweed extract (SWE) on the stem length (m) and stem diameter (cm) of *Azadirachta indica* seedlings 9 and 21 months after planting under treated wastewater irrigation. Each value represents the mean of independent replicates ± standard deviation. Different letter represent significant difference among treatments.

Biostimulant treatments significantly improved vegetative growth of *A. indica* seedlings compared with the untreated trees (Fig. 1). Seaweed extract at 2 g L^−1^ produced the greatest growth responses, followed closely by humic acid at 2 mL L^−1^.

Both seaweed extract and humic acid led to significant increments in stem length and diameter (*p* < 0.001 at 9 months; *p* = 0.0012 and 0.0219 at 21 months, respectively). Seaweed extract at 2 g L^−1^ resulted in the highest stem length (1.40 and 3.03 m), followed by humic acid at 2 mL L^−1^ (1.40 and 2.89 m), while the control recorded the lowest values (1.30 and 2.73 m) at 9 and 21 months, respectively. A similar trend was observed for stem diameter. Seaweed extract at 2 g L^−1^ produced the largest diameters (3.15 and 3.95 cm), followed by humic acid at 2 mL L^−1^ (3.12 and 3.74 cm), whereas the control showed the smallest diameters (2.41 and 3.54 cm) at 9 and 21 months after planting.

**Fig. 2:**
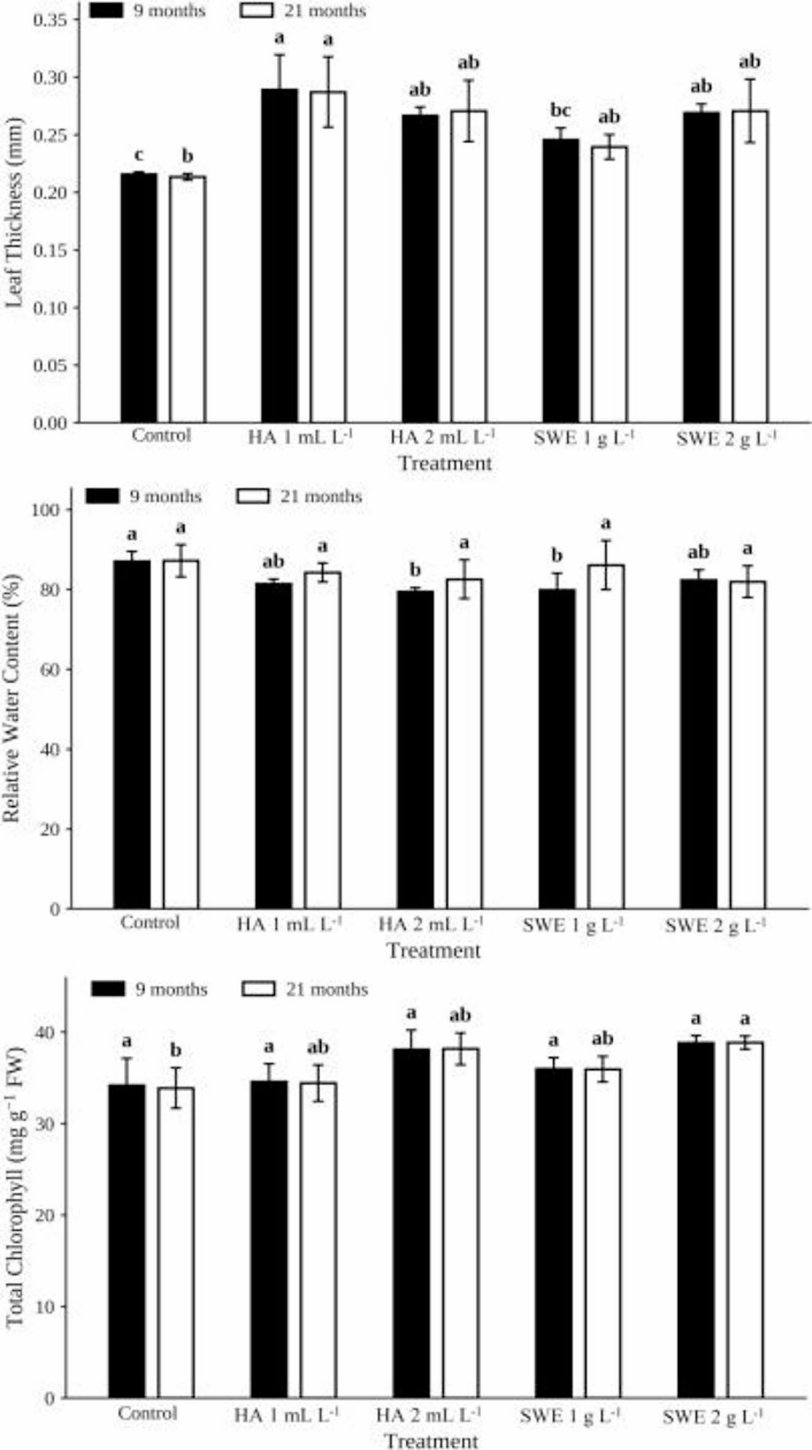
Effect of humic acid and seaweed extract on leaf thickness (mm), leaf water content (%), and total chlorophyll (mg m^−2^) of *Azadirachta indica* seedlings 9 and 21 months after planting under treated wastewater irrigation. Each value represents the mean of independent replicates ± standard deviation. Different letter represent significant difference among treatments.

Leaf morphological traits also responded to treatments; humic acid at 1 mL L^−1^ produced the thickest leaves (0.289 and 0.287 mm with *p* = 0.0014 and 0.016 at 9 and 21 months, respectively), while the control exhibited the thinnest leaves (0.216 and 0.213 mm). In contrast, leaf relative water content (RWC) was significantly influenced by treatments, *p* = 0.0299 at 9 months and 0.5717 at 21 months (Fig. 2). Control plants recorded the highest RWC (87.2% and 87.3% at 9 and 21 months), whereas seaweed extract at 2 g L^−1^ resulted in the lowest values (81.9% and 81.5%).

Total chlorophyll content was significantly affected by biostimulant application, *p* = 0.0538 and 0.0169 at 9 and 21 months, respectively (Fig. 2). Chlorophyll levels remained relatively stable across sampling periods. The control showed a slight decrease (177.3 to 174.6 mg m^−2^), whereas seaweed extract at 2 g L^−1^ maintained consistently high values (221.6 and 221.7 mg m^−2^), indicating sustained photosynthetic capacity in treated plants.

### Heavy metals (mg kg^−1^)

**Fig. 3:**
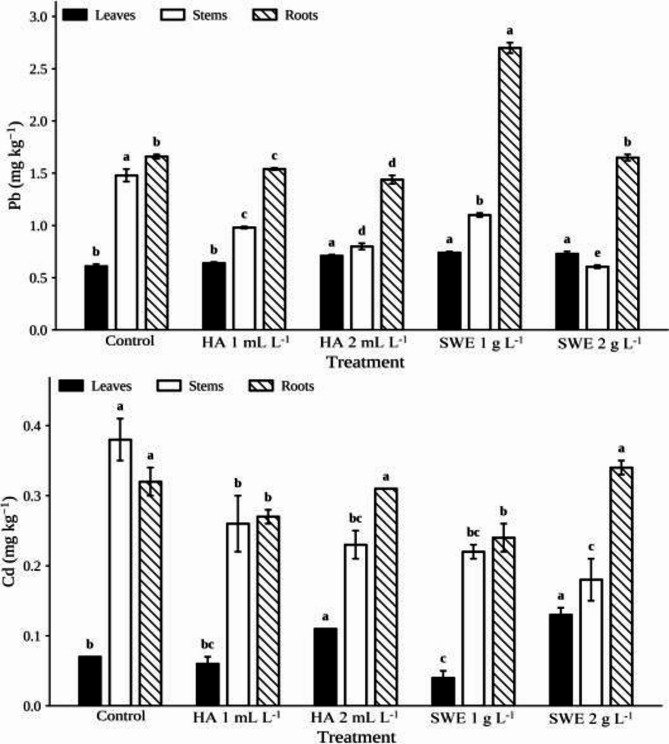
Effect of humic acid and seaweed extract on Pb and Cd (mg kg^−1^) contents in different parts of *Azadirachta indica* seedlings under treated wastewater irrigation. Each value represents the mean of independent replicates ± standard deviation. Different letter represent significant difference among treatments.

Biostimulants significantly (*P* < 0.001) influenced the accumulation of Pb, Cd, Cu, Fe, and Zn in leaves, stems, and roots of neem seedlings irrigated with treated wastewater. The highest Pb concentrations in roots (2.70 mg kg^−1^) and leaves (0.74 mg kg^−1^) were recorded with humic acid at 2 mL L^−1^, while the control produced the highest Pb level in stems (1.48 mg kg^−1^). Cadmium accumulation was greatest in stems of control plants (0.38 mg kg^−1^), whereas seaweed extract at 2 g L^−1^ resulted in the highest Cd concentrations in roots (0.34 mg kg^−1^) and leaves (0.13 mg kg^−1^). The lowest Cd value (0.04 mg kg^−1^) occurred in leaves treated with humic acid at 2 mL L^−1^ (Fig. 3).

**Fig. 4:**
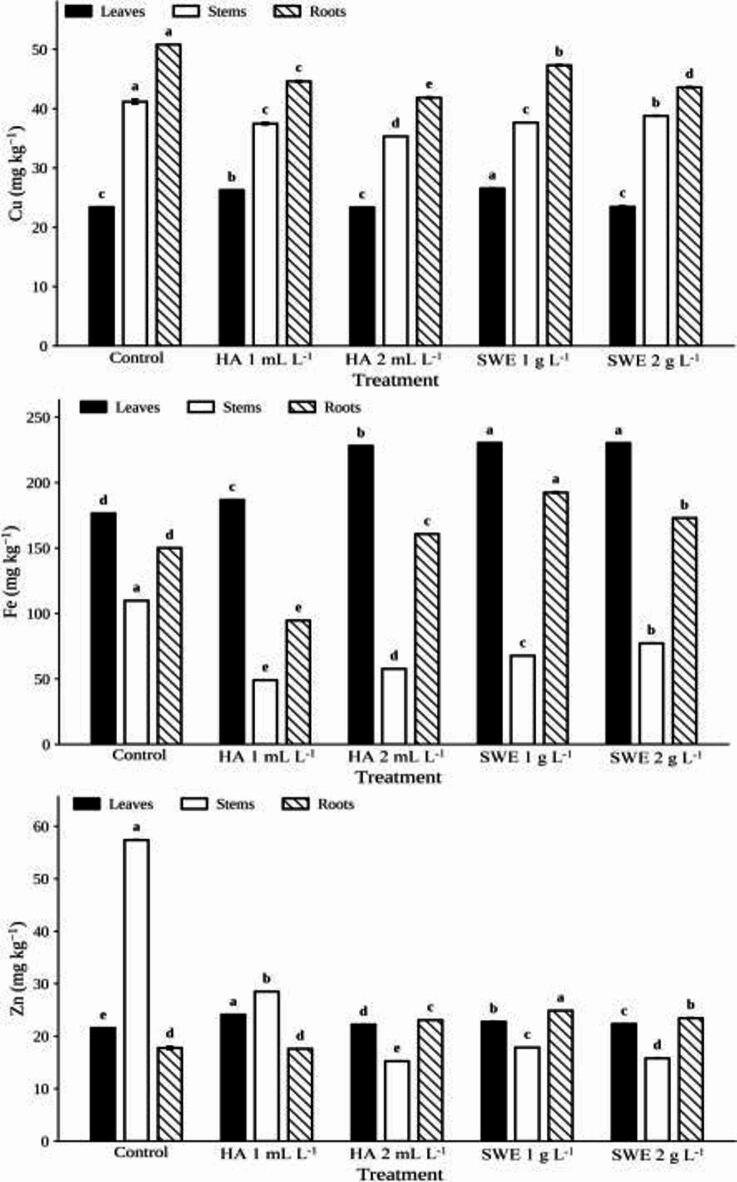
Effect of humic acid and seaweed extract on Cu, Fe and Zn (mg kg^−1^) contents in different parts of *Azadirachta indica* seedlings under treated wastewater irrigation. Each value represents the mean of independent replicates ± standard deviation. Different letter represent significant difference among treatments.

Copper concentrations were highest in roots (50.82 mg kg^−1^) and stems (41.20 mg kg^−1^) of control plants, while the highest leaf Cu content (26.57 mg kg^−1^) occurred with humic acid at 2 mL L^−1^. Seaweed extract at 1 g L^−1^ produced the lowest Cu levels across plant organs. Humic acid at 2 mL L^−1^ also produced the highest Fe concentrations in leaves (230.40 mg kg^−1^) and roots (192.50 mg kg^−1^). Zinc accumulation generally followed the order roots > stems > leaves. The highest root Zn concentration was recorded with humic acid at 2 mL L^−1^, while untreated plants showed the highest stem Zn level (Fig. 4).

### Physical and chemical wood properties

**Fig. 5:**
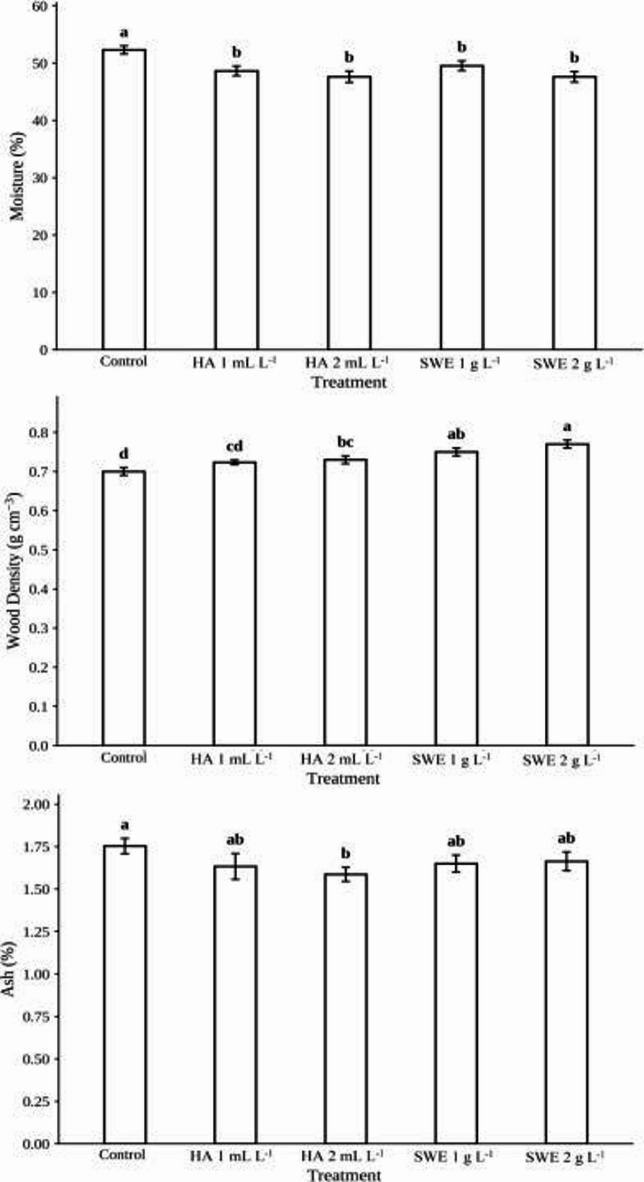
Effect of humic acid and seaweed extract on moisture content (%), wood density (g cm^−3^), and ash content (%) of *Azadirachta indica* wood under treated wastewater irrigation. Each value represents the mean of independent replicates ± standard deviation. Different letter represent significant difference among treatments.

Both biostimulants significantly affected the physical properties of neem wood (Fig. 5). All treatments reduced wood moisture content compared with the control (*p* < 0.001). The lowest moisture content (47.60%) was recorded with seaweed extract at 2 g L^−1^ and humic acid at 2 mL L^−1^. In contrast, wood density increased with biostimulant application, reaching the highest value with seaweed extract at 2 g L^−1^ (0.77 g cm^−3^; *p* < 0.001), compared with 0.70 g cm^−3^ in the control.

**Fig. 6:**
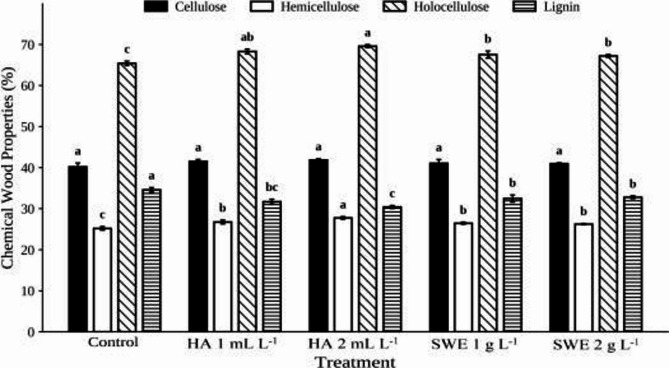
Effect of humic acid and seaweed extract on some chemical wood properties of *Azadirachta indica.* Each value represents the mean of independent replicates ± standard deviation. Different letter represent significant difference among treatments.

Chemical analysis (Fig. 6) showed that biostimulant treatments increased cellulose and hemicellulose contents, particularly with humic acid at 2 mL L^−1^ (41.82%; *p* = 0.083 cellulose and 27.77%; *p* < 0.001 hemicellulose). Consequently, holocellulose content increased to 69.58% compared with 65.40% in the control (*p* < 0.001). In contrast, lignin and ash contents were highest in untreated plants (34.60%; *p* < 0.001 and 1.75%; *p* = 0.0429) and decreased with biostimulant application, reaching their lowest values under humic acid at 2 mL L^−1^ (30.42% and 1.59%).

Overall, humic acid, especially at 2 mL L^−1^, enhanced structural holocellulose while reducing lignin and ash, indicating improved wood chemical quality.

## DIscussion

### Effects of heavy metal accumulation and biostimulants on plant growth and physiological responses

Extensive accumulation of elements in plant tissues has diverse effects on plant growth and physiological metabolism, whether toxic non-essential metals or essential micronutrients. Lead (Pb) is a non-essential toxic metal that impairs photosynthesis, chlorophyll biosynthesis, nutrient uptake, respiration, and enzyme activities, leading to oxidative stress, membrane damage, and reduced plant growth. Plants mitigate Pb toxicity by enhancing antioxidant defenses, producing metal-chelating compounds, and sequestering Pb into vacuoles and cell walls^10,12^. Also, cadmium (Cd) is a non-essential, highly phytotoxic metal that inhibits photosynthesis, nutrient uptake, respiration, and nitrogen metabolism while inducing oxidative stress and reducing plant growth and productivity. Plants tolerate Cd stress by activating antioxidant systems, synthesizing, and compartmentalizing Cd into vacuoles to limit cellular damage^10,12^.

On the other hand, copper (Cu) is an essential micronutrient required for normal plant growth and development; however, excessive Cu accumulation in contaminated soils becomes phytotoxic, inhibiting plant growth, reducing crop quality, and compromising the medicinal properties of plants. To mitigate Cu toxicity, plants employ adaptive mechanisms including limiting Cu uptake through root barriers, accumulating protective metabolites, and activating physiological and molecular defense responses that reduce Cu-induced oxidative damage^11,46^. Iron (Fe) is also an essential micronutrient that promotes chlorophyll biosynthesis, photosynthetic electron transport, chloroplast function, and key metabolic processes, including respiration, nitrogen metabolism, antioxidant defense, and redox homeostasis, thereby enhancing plant growth, productivity, and tolerance to environmental stresses^47,48^. However, both Fe deficiency and excess impair plant performance; deficiency disrupts chlorophyll synthesis and photosynthetic metabolism, whereas excess Fe induces ROS formation through Fenton reactions, causing oxidative stress, membrane damage, enzyme inhibition, and reduced plant productivity^47,49^.

In addition, zinc is an essential micronutrient involved in plant growth, nucleic acid metabolism, protein synthesis, membrane stability, hormone biosynthesis, and photosynthesis. It plays vital structural and regulatory roles in RNA and DNA while enhancing antioxidant defenses against ROS. Zinc also contributes to abiotic stress tolerance by regulating proline metabolism and activating antioxidant enzymes such as Cu-Zn superoxide dismutase, which detoxifies superoxide radicals and reduces oxidative damage under stress conditions^50–52^. Zinc deficiency weakens ROS scavenging systems, increasing oxidative stress, whereas excessive zinc inhibits cell division and elongation, reducing biomass production^53^. High zinc concentrations can also disrupt soil microbial diversity, decrease metal bioavailability, and impair soil fertility, crop quality, plant growth, and overall agricultural productivity^25,54,55^.

Biostimulants have been widely reported to enhance plant growth and physiological processes^24,56,57^. Humic acids convert minerals into organic forms that plants can easily absorb. Their main effects include improved root growth and morphology, enhanced nutrient uptake and utilization efficiency, and overall better plant growth through influence physiological processes such as photosynthesis, carbohydrate and protein synthesis, enzyme activity, and nucleic acid formation^24,58^. Similar benefits of humic acids in improving plant growth under adverse conditions^17,21^. In addition, seaweed extracts from marine macroalgae have been increasingly used due to their beneficial properties^25,57^. These extracts act as natural substances that reduce abiotic stress and enhance plant growth and productivity. They are obtained from various macroalgal species, producing complex mixtures of bioactive compounds depending on the extraction method^59^. Macroalgae are also effective biostimulants for plants grown under stress conditions^60^. Similar positive effects have been reported for several plants, including *Calotropis procera*^61^, *Moringa oleifera*^62^, and peach seedlings^63^.

In this study, biostimulants, particularly the higher concentration of seaweed extract, had a positive effect on the growth and physiological characteristics of neem trees irrigated with treated wastewater. The results showed that seaweed extract at 2 g L^−1^ increased plant height by 7.7% and 11.3%, stem diameter by 35.4% and 10.7%, and leaf thickness by 24.7% and 26.9%, accompanied by increases in total chlorophyll content of 13.6% and 14.7% during the first and second years, respectively. Notably, the increase in total chlorophyll content was positively associated with Fe accumulation in the leaves, reflecting the key role of Fe in chlorophyll synthesis and photosynthetic process^47,48^. In contrast, untreated plants exhibited slightly higher leaf relative water content; this does not necessarily indicate superior water status. Biostimulant-treated plants likely exhibited enhanced stomatal regulation, greater transpiration efficiency, improved osmotic adjustment, and increased biomass production, resulting in relatively lower leaf water content despite improved overall physiological performance. Similar responses have been reported in previous studies where enhanced metabolic activity reduced tissue water content while improving biomass^24,25,57^.

Biostimulants may act similarly to plant hormones, enhancing nitrogen uptake and plant performance, which increases biomass, leaf nitrogen, and chlorophyll content^64^. They also improve scion growth and chlorophyll concentration^65^. Positive effects on chlorophyll content and chemical composition were also observed in woody nursery trees such as *Cupressus × leylandii*, *Quercus virginiana*, and *Ulmus parvifolia*^66^.

### Heavy metal accumulation and phytoremediation potential of neem trees

Although the analysis of the treated wastewater indicated that the concentrations of heavy metals were within the permissible limits (Table 2), this does not necessarily imply that neem trees were not subjected to heavy metal stress. Even when present at low concentrations, heavy metals can gradually accumulate in the soil under long-term irrigation with treated wastewater, as occurred in the present study over approximately two years. On the other hand, soil analysis revealed a reduction in heavy metal concentrations after 21 months of cultivation compared with the initial pre-planting levels (Table 1). It is important to note that this reduction does not merely represent the difference between the initial and final soil concentrations; it also accounts for the continuous input of heavy metals introduced through treated wastewater irrigation throughout the 21-month experimental period. Therefore, the observed decline in soil heavy metal concentrations, despite the ongoing external inputs, strongly suggests that neem trees effectively absorbed and accumulated these metals, irrespective of the biostimulant treatments. These findings provide compelling evidence for the considerable phytoremediation potential of neem trees, highlighting their capacity to remove heavy metals from soils irrigated with treated wastewater over extended periods. Several studies proved the importance role of woody tree in phytoremediation and absorbtion of heavy metals from polluted water and soil^10–12^.

Phytoremediation is a sustainable approach that uses plants and their associated soil microorganisms to remove, immobilize, or reduce the toxicity of environmental pollutants. Plant species that absorb and accumulate high concentrations of metals in their above-ground biomass are suitable for phytoextraction, whereas species that limit metal mobility and bioavailability are effective for phytostabilization^10,67^. In this study, neem trees accumulated all the investigated heavy metals in different plant organs. The capacity of the plants to accumulate heavy metals varied depending on the biostimulant treatment. Notably, metal accumulation also differed according to the type of element, with most metals accumulating at higher levels in the roots than in the stems and leaves. Iron was the exception, showing greater accumulation in the leaves than in the stems and roots. These findings indicate that neem trees employ different phytoremediation mechanisms depending on the specific heavy metal.

Several studies have shown that biostimulants can reduce heavy metal toxicity in plants. For example, humic acid increased the adsorption capacity of mangrove sediments for Cd, Cu, and Pb^68^. In mangrove (*Avicennia germinans*) seedlings grown under Cd stress, humic acid improved root anatomy and architecture, enhancing root length and surface area and supporting phytoremediation processes^69^. Root growth stimulation generally occurs at low HA concentrations, whereas higher doses may inhibit growth^70^.

Seaweed extracts also act as agricultural biostimulants due to their bioactive polymers, which help plants cope with biotic and abiotic stresses^50,51,57,71^. Hu reported that a commercial seaweed biostimulant enhanced Cd uptake by roots of the accumulator *Nasturtium officinale* while reducing Cd translocation to shoots. Although this effect may limit its use for phytoremediation, it suggests potential benefits for cultivating crops in Cd-contaminated soils.

### Effects of heavy metal accumulation and biostimulants on wood properties

In this study, biostimulants significantly affected the physical and chemical wood properties of the studied tree species, with superior to seaweed extract. SWE at 2 g L^−1^ increased wood density by 10% compared to control. Meanwhile, HA at 2 ml L^−1^ decreased wood moisture content by 9%, ash by 9.5%, and lignin by 12.1%. In contrast, HA at 2 ml L^−1^ increased cellulose, hemicellulose and holocellulose by 4, 10.2, and 6.4% compared to control, respectively. Such variations may result from factors such as tree age and genotype, as well as geographic conditions including latitude, temperature, and precipitation^72^. These results proved the important role of seaweed extract not only in improving growth and physiological metabolism, but also for improving wood quality. In addition, the ability to obtain high wood quality by using irrigating woody trees with treated wastewater.

Regarding wood chemical composition, biostimulant treatments increased cellulose, hemicellulose, and holocellulose percentages compared with untreated plants. The highest values were recorded with HA at 2 ml L⁻¹, while lignin and ash contents were higher in the control. Wood chemical composition varies among species and growing conditions. In the present study, differences in chemical properties among tree species and treatments may be related to variations in growth rate. (Shanbhag and Sundararaj,^73^) reported that cellulose contents ranging from 43% in *Tectona grandis* (Myanmar) to 59% in *Shorea marcoptera* (Malaysia), while lignin ranged from 22% in *Fagus sylvatica* and *Acer pseudoplatanus* to 36% in *T. grandis*. Growth and wood characteristics may respond to fertilization for several years depending on fertilizer level and stand management.

## Conclusion

This study demonstrates that the application of biostimulants can substantially improve the performance of *Azadirachta indica* irrigated with treated wastewater while simultaneously enhancing its potential for sustainable environmental remediation. Among the tested treatments, seaweed extract at 2 g L^−1^ was the most effective in promoting vegetative growth, maintaining higher chlorophyll content, increasing wood density, and improving overall wood quality. In contrast, humic acid at 2 mL L^−1^ was particularly effective in enhancing cellulose, hemicellulose, and holocellulose contents while reducing lignin and ash, thereby improving the chemical quality of the wood. The results further confirm that neem trees effectively accumulate heavy metals from soils irrigated with treated wastewater, supporting their suitability as a phytoremediation species. Importantly, biostimulant application enhanced tree growth and wood quality without compromising this phytoremediation capacity, indicating that improved biomass production and environmental cleanup can be achieved simultaneously. These findings suggest that the integration of treated wastewater irrigation with appropriate biostimulant application represents a practical and sustainable strategy for establishing productive woody plantations in water-limited regions. Based on the experimental findings, seaweed extract at 2 g L^−1^ is recommended when the primary objective is to maximize tree growth and wood physical quality, whereas humic acid at 2 mL L^−1^ is preferable when improving wood chemical composition is the priority.

### Study limitations

This study did not evaluate physiological and biochemical stress indicators, such as proline, H₂O₂, malondialdehyde, or antioxidant enzyme activities. In addition, the study did not investigate the effect of different biostimulants on physical and chemical of soil. Including these parameters would provide a better understanding of the mechanisms by which humic acid and seaweed extract alleviate stress under long-term treated wastewater irrigation. Future studies should integrate these biomarkers with growth and heavy metal accumulation assessments.

## Data Availability

All needed data is combined in the main text, and any other information can be given by the corresponding author.
